# A 3-year follow-up study after treatment with simeprevir in combination with pegylated interferon-α and ribavirin for chronic hepatitis C virus infection

**DOI:** 10.1186/s12985-018-0936-4

**Published:** 2018-01-30

**Authors:** Fabien Zoulim, Christophe Moreno, Samuel S. Lee, Peter Buggisch, Andrzej Horban, Eric Lawitz, Chris Corbett, Oliver Lenz, Bart Fevery, Thierry Verbinnen, Umesh Shukla, Wolfgang Jessner

**Affiliations:** 1 INSERM U1052-CNRS 5286, Cancer Research Center of Lyon (CRCL) and Hepatology Department, Lyon, France; 2 CUB Hôpital Erasme, Université Libre de Bruxelles, Brussels, Belgium; 3 Liver Unit, University of Calgary, Calgary, AB Canada; 4 Institute for Interdisciplinary Medicine, Hamburg, Germany; 5 Department for Adult’s Infectious Diseases, Hospital for Infectious Diseases, HIV Out-Patient Clinic and Medical University of Warsaw, Warsaw, Poland; 6 Texas Liver Institute, University of Texas Health, San Antonio, TX USA; 7 Janssen Research & Development, Janssen Pharmaceutica NV, Beerse, Belgium; 8 Janssen Research & Development, LLC, Titusville, NJ USA

**Keywords:** Direct-acting antivirals, Hepatitis C virus, Pegylated interferon, Simeprevir, Sustained virologic response, NS3 amino acid substitutions

## Abstract

**Background:**

Simeprevir is approved with pegylated interferon and ribavirin (PR) for chronic hepatitis C virus (HCV) genotype (GT) 1 and GT4 infection in the USA and the European Union.

**Methods:**

This 3-year follow-up study assessed the durability of sustained virologic response (SVR) (undetectable HCV RNA 12 or 24 weeks after treatment end), and evaluated the persistence of treatment-emergent NS3/4A protease inhibitor resistance in patients not achieving SVR following treatment with simeprevir plus PR in the parent study. The maintenance of SVR after the last post-therapy follow-up visit of the parent study (LPVPS) was assessed using HCV RNA measurements. The persistence of treatment-emergent NS3 amino acid substitutions in patients with no SVR at LPVPS was assessed using population sequencing. No study medications were administered.

**Results:**

Overall, 249 patients were enrolled (200 with SVR at LPVPS; 49 with no SVR at LPVPS); 40 patients discontinued prematurely (18 with SVR; 22 with no SVR). All 200 enrolled patients who achieved SVR in the parent study maintained SVR until the last available visit in this study (median follow-up time: 35.8 months). The treatment-emergent NS3 amino acid substitutions detected at time of failure in the parent study in 43/49 enrolled patients were no longer detected in 37/43 (86.0%) at the end of this study (median follow-up time: 179.9 weeks [41.3 months]).

**Conclusion:**

This 3-year follow-up study provides evidence for the long-term durability of SVR (100%) after successful treatment with simeprevir plus PR. Treatment-emergent NS3 amino acid substitutions became undetectable in the majority of patients.

**Trial registration:**

NCT01349465; ClinicalTrials.gov.

**Electronic supplementary material:**

The online version of this article (10.1186/s12985-018-0936-4) contains supplementary material, which is available to authorized users.

## Background

Simeprevir – an oral, once-daily (QD) hepatitis C virus (HCV) NS3/4A protease inhibitor with antiviral activity against HCV genotype (GT) 1, 2, 4, 5 and 6 [1–4] – is approved in combination with pegylated interferon and ribavirin (PR) for chronic HCV GT1 and GT4 infection, with or without human immunodeficiency virus (HIV) co-infection, in the USA and the European Union (EU) [5, 6]. In addition, simeprevir is approved as part of an interferon (IFN)-free combination with sofosbuvir (a QD pangenotypic HCV nucleotide-analogue NS5B polymerase inhibitor) in the USA for HCV GT1 infection, and in the EU for HCV GT1, 4 and HCV/HIV coinfection [5, 6].

Achieving sustained virologic response (SVR) 12 weeks after the end of treatment (SVR12) is a well-established surrogate marker for cure following HCV treatment, and has previously been defined as undetectable HCV RNA levels and, more recently, as HCV levels below the lower limit of quantification (LLOQ). It is well supported that patients benefit from achieving SVR. In a meta-analysis of 31 studies evaluating treatment for chronic HCV infection, the pooled 5-year mortality rates for patients achieving SVR were significantly lower than those for patients not achieving SVR in the general population, in patients with cirrhosis and in patients co-infected with HIV [7].

Factors that may be associated with virologic failure include the presence, at pre-treatment, of resistance-associated substitutions (RASs) within the viral genes targeted by the direct-acting antiviral (DAA) components of the regimen [8]. In the virology analyses of the Phase IIb or Phase III simeprevir plus PR studies, it was concluded that NS3 amino acid substitutions emerging at the time of failure were no longer detectable by the end of the study in 50.0% [90/180] of patients analysed by population sequencing [9]. In contrast, long-term persistence has been demonstrated for NS5A amino acid substitutions that emerged in patients who failed an NS5A inhibitor-containing regimen, [10] which may impact on re-treatment strategies.

This prospective, 3-year follow-up study was conducted to assess the durability of SVR and to evaluate the time to return to baseline sequence for the treatment-emergent NS3 amino acid substitutions in patients not achieving SVR following treatment with simeprevir plus PR. Liver disease evolution and safety results are also reported.

## Methods

### Patients and study design

This was a prospective, 3-year, multicentre study in patients who completed the last post-therapy follow-up visit (LPVPS) of a previous Phase IIb or Phase III study in which they had received simeprevir in combination with PR for the treatment of HCV infection. No study medication was administered in this follow-up study. Patients were enrolled at 50 sites in Belgium, Canada, France, Germany, Poland, the Russian Federation and the USA. Parent studies included two Phase IIb studies (ASPIRE [NCT00980330] [4] and PILLAR [NCT00882908]) [1] and three Phase III studies (QUEST-1 [NCT01289782] [11], QUEST-2 [NCT01290679] [12] and PROMISE [NCT01281839]) [13]. Inclusion criteria for these parent studies were standard for studies including treatment with PR, and the specifics can be found in the aforementioned published papers.

The study was approved by the relevant Institutional Review Board or Independent Ethics Committee at each study centre and met the principles of the Declaration of Helsinki and Good Clinical Practice guidelines. All patients provided informed, written consent to participate.

To be eligible for this follow-up study, patients had to have received at least one dose of simeprevir in combination with PR in one of the parent studies (75/100/150 mg simeprevir for durations of 12, 24 or 48 weeks dependent on the design of the parent study). Patients had to have completed LPVPS no longer than 6 months prior to enrolment into this follow-up study. Patients who received or were planning to receive antiviral or systemic immune-modulating treatment, or who participated in another study, were not eligible. Screening for eligible patients was performed at LPVPS or at any time between LPVPS and the Month 6 (relative to LPVPS) observation point of this follow-up study. The total study period for each patient was a maximum of 36 months.

### Study objectives

The co-primary objectives of this study were to evaluate the durability of SVR in patients who were treated with simeprevir in combination with PR in a previous Phase IIb or Phase III study and who had achieved SVR at LPVPS (will be referred to as: SVR patients), and to evaluate sequence changes in the HCV NS3/4A region over time in patients not having achieved SVR at LPVPS (will be referred to as: no-SVR patients).

The primary efficacy parameter for SVR patients was maintenance of SVR at each time point in this study, including the last available measurement. The last available measurement in this follow-up study was considered as the primary time point to evaluate the durability of SVR. In addition, in no-SVR patients, the persistence of treatment-emergent amino acid substitutions at 18 NS3 positions of interest (36, 41, 43, 54, 55, 80, 107, 122, 132, 138, 155, 156, 158, 168, 169, 170, 174 and 175) was determined over time. Of note, the planned resistance analysis in this study considered any amino acid substitution at the 18 NS3 positions of interest, and was not limited to simeprevir RASs (substitutions which confer a > 2-fold reduction in simeprevir activity in vitro) [14].

Secondary efficacy parameters included the proportion of patients with late viral relapse (i.e., SVR patients that subsequently experienced a viral relapse) as well as an evaluation of changes in the HCV NS3/4A sequence of patients with late viral relapse. Assessment of the development of liver disease evolution in patients previously treated with simeprevir plus PR was also a secondary objective; however, data collection was optional.

### Procedures

Blood samples for HCV RNA level determination were collected every 6 months up to 36 months after LPVPS. HCV RNA was measured using the Roche COBAS® TaqMan® HCV Test v2.0 for use with the High Pure System (with an LLOQ of 25 IU/mL and limit of detection of 10–15 IU/mL).

Samples for viral sequencing were taken every 6 months (relative to LPVPS), and the HCV NS3/4A region was sequenced using population sequencing in all enrolled no-SVR patients.

All adverse events (AEs) were monitored throughout the study until Month 36. AEs were coded according to Medical Dictionary for Regulatory Activities preferred terms (version 14.1), with severity being determined according to the World Health Organization toxicity grading scale. The relationship of AEs to study treatment was assessed by the investigator. Laboratory parameters were also reported.

Liver disease progression was an optional assessment performed in some patients by liver biopsy or alternative method (including FibroScan). Conversion to a METAVIR score was performed at the site by the investigator.

### Statistical analysis

All patients who signed the informed consent form were included in the analysis. No formal power calculation was performed.

As this was a 3-year follow-up study to assess the sustainability for SVR patients, and the viral resistance in the no-SVR patients, all analyses were conducted descriptively without statistical hypothesis testing.

For the primary efficacy endpoints, if a time point was missing, but there was a measurement available at a later time window, then the measurement at the later time point was used. If there was > 1 measurement at the later time window, then the first measurement was used. The secondary endpoints included late viral relapse and safety, which were summarized descriptively.

## Results

### Patients

**Fig. 1:**
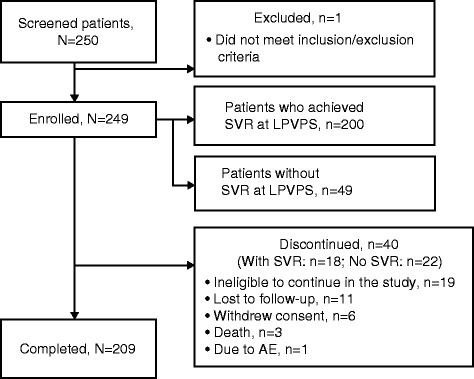
Patient disposition. *AE* adverse event, *LPVPS* last post-therapy follow-up visit of the parent study, *SVR* sustained virologic response

Patient disposition is shown in Fig. 1. Of the 250 patients screened, 249 were enrolled; 64.3% of patients (160/249) were from ASPIRE [4], 14.1% (35/249) from PILLAR [1], 5.6% (14/249) from QUEST-1 [11], 6.4% (16/249) from QUEST-2 [12] and 9.6% (24/249) from PROMISE [13]. Of the 249 patients, 200 were SVR patients and 49 were no-SVR patients. Forty patients discontinued the study prematurely (18 SVR patients and 22 no-SVR patients): 19 no-SVR patients were ineligible to further continue the study (16 patients started using disallowed medication; 3 patients enrolled in a clinical study with an investigational drug or investigational medical device), 11 were lost to follow-up (10 SVR; 1 no-SVR), 6 withdrew consent (4 SVR; 2 no-SVR), 3 SVR patients died (1 of cholangitis and pancreatic carcinoma, 1 of myocardial infarction [not related to parent study drugs] and 1 of malignant hepatic neoplasm [doubtfully related to parent study drugs]), and 1 SVR patient discontinued due to an AE. Patients who discontinued prematurely had a median follow-up time of 25.2 months.

Baseline demographic and disease characteristics are presented in Table 1. The patients enrolled in this study were a representative subset of the patients in the parent studies. The majority of patients were male (61.8%) and white (92.8%), and the median age was 54.0 (range: 22.0–70.0) years. Overall, *interleukin-28B* (*IL28B*) CC, CT and TT genotypes were observed in 18.5% (10/54), 66.7% (36/54) and 14.8% (8/54) of patients, respectively (data not available for patients from the PILLAR and ASPIRE studies). Of the 68 enrolled HCV GT1a-infected SVR patients, 21 (30.9%) carried a NS3 Q80K polymorphism at baseline. A NS3 Q80K polymorphism was observed in 45.4% (79/174) of the eligible and 40.0% (10/25) of the enrolled HCV GT1a-infected no-SVR patients.

**Table 1:** Demographics and baseline disease characteristics

	SVR at LPVPS	No SVR at LPVPS	
	*N* = 200	Eligible *N* = 294	Enrolled *N* = 49
Age, years; median (range)	52.0 (22–70)	53.0 (19–71)	56.0 (28–70)
≤ 45 years, n (%)	61 (30.5)	64 (21.8)	7 (14.3)
> 45 − ≤ 65 years, n (%)	133 (66.5)	218 (74.1)	38 (77.6)
> 65 years, n (%)	6 (3.0)	12 (4.1)	4 (8.2)
Male, n (%)	122 (61.0)	196 (66.7)	32 (65.3)
Ethnicity, n (%)			
Hispanic or Latino	7 (3.5)	35 (11.9)	5 (10.2)
Race, n (%)^a^			
White	187 (93.5)	268 (91.2)	44 (89.8)
Black/African American	8 (4.0)	21 (7.1)	4 (8.2)
American Indian/Alaskan native	1 (0.5)	0	0
Asian	4 (2.0)	3 (1.0)	1 (2.0)
Native Hawaiian or other Pacific islander	0	2 (0.7)	0
*IL28B* genotype, n/N (%)^b^			
CC	10/34 (29.4)	9/143 (6.3)	0
CT	19/34 (55.9)	99/143 (69.2)	17/20 (85.0)
TT	5/34 (14.7)	35/143 (24.5)	3/20 (15.0)
Prior response, n (%)^a^			
Naïve	50 (25.0)	138 (46.9)	15 (30.6)
Null responder	26 (13.0)	46 (15.6)	12 (24.5)
Partial responder	48 (24.0)	38 (12.9)	9 (18.4)
Relapser	76 (38.0)	72 (24.5)	13 (26.5)
Baseline HCV RNA, log_10_ IU/mL; median (range)^a^	6.44 (3.5–7.5)	6.62 (4.9–7.6)	6.76 (5.6–7.5)
LPVPS HCV RNA, log_10_ IU/mL; median (range) ^a,c^	0.95 (1.0–1.0)	6.35 (3.2–7.4)	6.35 (3.2–7.4)
HCV geno/subtype^a^			
1a/other	68 (34.0)	174 (59.2)	25 (51.0)
with Q80K	21 (30.9)	79 (45.4)	10 (40.0)
1b	132 (66.0)	120 (40.8)	24 (49.0)
METAVIR fibrosis score; n/N (%)^a,d^			
F0, 1 or 2	152/199 (76.4)	156/290 (53.8)	27/49 (55.1)
F3	26/199 (13.1)	76/290 (26.2)	8/49 (16.3)
F4	21/199 (10.6)	58/290 (20.0)	14/49 (28.6)

*HCV* hepatitis C virus, *IL28B* interleukin-28b, *LPVPS* last post-therapy follow-up visit of the parent study, *SVR* sustained virologic response

^a^Obtained from the parent study

^b^Results obtained from the central laboratory in parent study; data not available for patients from the PILLAR and ASPIRE studies

^c^Undetectable HCV RNA is imputed with 9 IU/mL (log_10_[9] = 0.95)

^d^METAVIR score was not available for one patient

### Efficacy

#### Sustained virologic response

The durability of SVR was evaluated in all enrolled SVR patients. All 200 patients maintained SVR until the last available visit. The median follow-up time was 35.8 months (range: 6.7–38.4 months).

Late viral relapse was evaluated in all enrolled SVR patients. All patients maintained SVR until the last available visit; therefore, no late viral relapse was observed.

#### Resistance determination

**Fig. 2:**
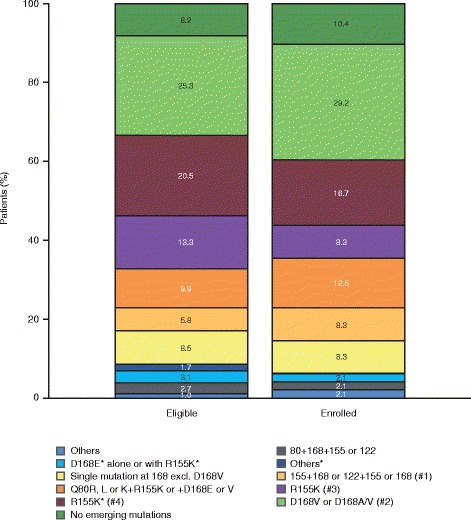
Emerging NS3 amino acid substitutions at time of failure in simeprevir Phase IIb/III studies. Considering emerging amino acid substitutions at 18 NS3 positions of interest: 36, 41, 43, 54, 55, 80, 107, 122, 132, 138, 155, 156, 158, 168, 169, 170, 174 and 175. One patient had no sequencing data available. *Amino acid substitutions in patients with Q80K at baseline. #1: R155K or Q + D168E/V or A/V or S122R or G + R155K or D168A, includes one patient with I132L + R155K + D168E + N174G; #2: includes one patient with V132I + D168V. #3: includes one patient with V36M + R155K; #4: alone or in combination with I132L*, I170T* or N174S*

Paired baseline and time-of-failure sequencing information was available for 48 of the 49 enrolled no-SVR patients. The subset of enrolled no-SVR patients was representative of all eligible patients from the parent studies, with regards to baseline characteristics and emerging NS3 amino acid substitutions at time of failure (when considering the 18 NS3 positions of interest; Fig. 2). Of the 269/293 eligible patients with emerging NS3 amino acid substitutions at time of failure in the parent studies, 137 (50.9%) returned to baseline at the end of the parent study.

The number of enrolled no-SVR patients who had emerging NS3 amino acid substitutions at the time of failure in the parent study, and returned to the baseline sequence or had a change in NS3 amino acid substitution profile at the end of this study, is presented in Table 2. In total, 43/48 enrolled patients had emerging NS3 amino acid substitution at time of failure in the parent study, which returned to baseline sequence in 24/43 patients (55.8%) at the end of the parent study (median follow-up time: 35.4 weeks [range: 5.9–69.9 weeks]). In an additional 13/43 patients, the HCV sequence returned to baseline at the end of this follow-up study, resulting in a total of 37/43 (86.0%) enrolled no-SVR patients who lost the emerging NS3 amino acid substitutions observed at time of failure in the parent study (median follow-up time: 179.9 weeks [41.3 months; range: 46.7–230.3 weeks]). For HCV GT1a-infected patients, the median follow-up time was 182.1 weeks (range: 96.1–230.3 weeks) and for HCV GT1b-infected patients this was 174.9 weeks (range: 46.7–225.1 weeks).

**Table 2:** Amino acid substitutions in 18 NS3 positions of interest

NS3 amino acid profile at time of failure^a^	N	Return to baseline at EOS^a,c,d^, n (%)	Change to new profile at EOS^a,c^, n (%)	Follow-up time^b^, median, weeks	Follow-up time^b^, range, weeks
*All HCV geno/subtypes*					
Enrolled patients with no SVR at LPVPS	49				
Number of patients (at failure with EOS) with sequencing information^c^	48	37 (77.1)	3 (6.3)	177.7	(46.7–230.3)
No emerging NS3 amino acid substitution	5	0 (0.0)	0 (0.0)	174.0	(111.3–192.0)
Any emerging NS3 amino acid substitution	43	37 (86.0)	3 (7.0)	179.9	(46.7–230.3)
*HCV GT1a with Q80K at baseline*					
Enrolled patients with no SVR at LPVPS	10				
Number of patients (at failure with EOS) with sequencing information^c^	10	8 (80.0)	0 (0.0)	146.9	(96.1–230.3)
No emerging NS3 amino acid substitution	1	0 (0.0)	0 (0.0)	111.3	–
Any emerging NS3 amino acid substitution	9	8 (88.9)	0 (0.0)	180.9	(96.1–230.3)
R155K	8	8 (100.0)	0 (0.0)	146.9	(96.1–230.3)
D168E	1	0 (0.0)	0 (0.0)	192.4	–
*HCV GT1a without Q80K at baseline*					
Enrolled patients with no SVR at LPVPS	14				
Number of patients (at failure with EOS) with sequencing information^c^	14	12 (85.7)	1 (7.1)	182.6	(98.9–222.0)
Any emerging NS3 amino acid substitution	14	12 (85.7)	1 (7.1)	182.6	(98.9–222.0)
R155K	4	3 (75.0)	0 (0.0)	179.4	(98.9–198.0)
D168V	2	2 (100.0)	0 (0.0)	141.6	(138.0–145.1)
Q80R + D168E	2	2 (100.0)	0 (0.0)	214.4	(210.0–218.7)
R155K + D168E	2	1 (50.0)	1 (50.0)	182.6	(182.1–183.0)
D168A	1	1 (100.0)	0 (0.0)	134.6	–
D168E	1	1 (100.0)	0 (0.0)	192.0	–
R155K + D168A	1	1 (100.0)	0 (0.0)	189.0	–
R155K + D168V	1	1 (100.0)	0 (0.0)	222.0	–
*HCV GT1b*					
Enrolled patients with no SVR at LPVPS	24				
Number of patients (at failure with EOS) with sequencing information^c^	24	17 (70.8)	2 (8.3)	174.9	(46.7–225.1)
No emerging NS3 amino acid substitution	4	0 (0.0)	0 (0.0)	175.3	(157.1–192.0)
Any emerging NS3 amino acid substitution	20	17 (85.0)	2 (10.0)	174.9	(46.7–225.1)
D168V	10	10 (100.0)	0 (0.0)	148.5	(46.7–225.1)
Q80R + D168E	2	0 (0.0)	1 (50.0)	169.5	(126.9–212.1)
D168A	1	1 (100.0)	0 (0.0)	188.4	–
D168E	1	1 (100.0)	0 (0.0)	169.3	–
D168E/V	1	1 (100.0)	0 (0.0)	220.3	–
Q80K + D168E	1	1 (100.0)	0 (0.0)	174.6	–
Q80K + S122R + D168E	1	0 (0.0)	1 (100.0)	175.9	–
Q80R + D168E/V	1	1 (100.0)	0 (0.0)	196.3	–
Q80R + S174F/Y	1	1 (100.0)	0 (0.0)	166.4	–
V132I + D168V	1	1 (100.0)	0 (0.0)	221.0	–

The above table presents return-to-baseline^a,c,d^ or change to new^a,c^ amino acid substitution in 18 NS3 positions of interest^a^ at the end of this follow-up study, by HCV genotype/subtype and presence of baseline NS3 Q80K, in patients with no SVR at LPVPS

*EOS* end of study, *GT* genotype, *HCV* hepatitis C virus, *LPVPS* last post-therapy follow-up visit of the parent study, *SVR* sustained virologic response

^a^Only emerging NS3 amino acid substitutions at 18 selected positions (36, 41, 43, 54, 55, 80, 107, 122, 132, 138, 155, 156, 158, 168, 169, 170, 174,175) were considered

^b^Follow-up time is the time (weeks) between the date of the last available sample in this study and the time of failure sample from the parent study

^c^EOS: last available sequencing sample from this study

^d^Return to baseline: return to amino acid substitutions that were present at baseline, or to wildtype

In total, 6/43 patients (three patients each with GT1a and GT1b) still had emerging NS3 amino acid substitutions present at the end of the parent study that persisted at the end of this follow-up study, five of whom completed this study and one who had follow-up data up to Month 24. Three out of these six patients had the same emerging NS3 amino acid substitutions at the end of this study as were present at time of failure in the parent study (Q80R + D168E, R155K and D168E). For the three other patients, a change in NS3 amino acid substitution profile at the end of this study compared with the time of failure in the parent study was found: one patient with S122R + D168E at end of this study compared with Q80K + S122R + D168E at time of failure, a second with Q80K at end of this study compared with Q80R + D168E at the time of failure, and a third with R155K + D168E + I170V at the end of this study compared with R155K + D168E at the time of failure. Note that I170V does not reduce simeprevir in vitro activity [9].

Of note, none of the five patients without emerging NS3 amino acid substitutions at the time of failure in the parent study, and who had completed this study, had emerging amino acid substitutions at any of the 18 NS3 positions of interest at the end of this study, or at any time during follow-up.

**Fig. 3:**
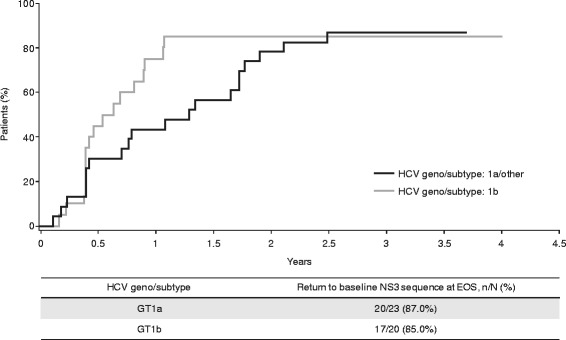
Time to return to baseline NS3 sequence* in the parent Phase IIb/III studies. *From time of failure; considering the 18 NS3 positions of interest: 36, 41, 43, 54, 55, 80, 107, 122, 132, 138, 155, 156, 158, 168, 169, 170, 174 and 175. *EOS* end of study, *GT* genotype, *HCV* hepatitis C virus

The median time to return to baseline sequence was less for HCV GT1b-infected patients than for HCV GT1a/other-infected patients (30.5 vs 67.4 weeks) (Fig. 3). Among HCV GT1a/other-infected patients, the median time to return to baseline was less in those with Q80K at baseline than in those without (36.4 vs 89.9 weeks; data not shown). Patients with Q80K at baseline had emerging R155K or D168E NS3 amino acid substitutions at time of failure.

### Liver disease evolution

Assessment of liver disease evolution was a secondary objective in this study and data collection was optional. Please see Additional file 1 for results of the liver disease evolution analysis.

### Clinical outcomes

A summary of clinical outcomes by SVR status at LPVPS is presented in Table 3. During the 36-month study period, AEs were reported in 11/249 (4.4%) patients (SVR: 10/200 [5.0%]; no-SVR: 1/49 [2.0%]); three of these AEs (1.2%) were Grade 1 or 2, and eight (3.2%) were Grade 3 or 4. Serious AEs (SAEs) were reported in 10/249 (4.0%) patients (all with SVR), and none were considered by the investigator to be related to study treatment. In 2/8 (25.0%) patients on disallowed medication, two AEs were reported: one Grade 2 and one Grade 3. In addition, in 1/8 (12.5%) patients on disallowed medication, an SAE unrelated to study treatment was reported.

**Table 3:** Summary of AEs

	SVR at LPVPS *N* = 200	No SVR at LPVPS *N* = 49	Total *N* = 249
*36-month study period*			
Any AE, n (%)	10 (5.0)	1 (2.0)	11 (4.4)
Worst Grade 1 or 2	2 (1.0)	1 (2.0)	3 (1.2)
Worst Grade 3 or 4	8 (4.0)	0	8 (3.2)
Any SAE, n (%)	10 (5.0)	0	10 (4.0)
SAE at least possibly related to the study drug	0	0	0
Any AE with a fatal outcome, n (%)	3 (1.5)^a^	0	3 (1.2)^a^
AE for which study procedure was permanently stopped, n (%)	1 (0.5)^b^	0	1 (0.4)^b^
AE of hepatocellular carcinoma type, n (%)	3 (1.5)	0	3 (1.2)
*Patients on disallowed medication* ^*c*^	*n* = 0	*n* = 8	*n* = 8
Any AE, n (%)			
Worst Grade 1 or 2	0	1 (12.5)	1 (12.5)
Worst Grade 3 or 4	0	1 (12.5)	1 (12.5)
Any SAE, n (%)	0	1 (12.5)	1 (12.5)
SAE at least possibly related to the study drug	0	0	0
Any AE with a fatal outcome, n (%)	0	0	0
AE for which study procedure was permanently stopped, n (%)	0	0	0
AE of hepatocellular carcinoma type, n (%)	0	1 (12.5)	1 (12.5)

*AE* adverse event, *HCV* hepatitis C virus, *LPVPS* last post-therapy follow-up visit of the parent study, *SAE* serious adverse event, *SVR* sustained virologic response

^a^Grade 4 cholangitis and pancreatic carcinoma, Grade 3 hepatic neoplasm malignant and Grade 4 myocardial infarction

^b^Grade 3 hepatic neoplasm malignant

^c^AEs reported for patients who started disallowed HCV medication are presented separately from the time this medication was started

During the 36-month study period, one patient discontinued the study due to an AE that was not considered to be related to the parent study drug. The most frequently reported AEs were in the system organ classes: neoplasms benign, malignant and unspecified (including cysts and polyps) (6/249 patients [2.4%]), and hepatobiliary disorders (3/249 patients [1.2%]). Three no-SVR patients had AEs with fatal outcomes. One patient had both cholangitis and pancreatic carcinoma (both considered to be not related to the parent study drug), one had a myocardial infarction (considered to be not related to the parent study drug) and one had malignant hepatic neoplasm (considered to be doubtfully related to the parent study drug). During the 36-month study period, AEs related to hepatocellular carcinoma (HCC) were reported for three SVR patients, all of whom had cirrhosis. An additional no-SVR patient with cirrhosis was on disallowed medication and an AE of HCC was reported. Therefore, 4/35 (11.4%) enrolled patients with cirrhosis had an AE related to HCC in this study. All cases of HCC were de novo.

Of the clinical laboratory evaluations, mean haemoglobin, platelets, alkaline phosphatase, albumin, bilirubin levels and prothrombin time stayed constant throughout the 36-month study period. For SVR patients, mean platelet levels and albumin were slightly higher throughout, and the mean levels of bilirubin were slightly lower than for no-SVR patients. For haemoglobin, alkaline phosphatase and prothrombin time, mean levels stayed similar between the two groups.

Differences were observed for the levels of alanine aminotransferase and aspartate aminotransferase, as a consistent marked reduction was observed in SVR patients compared with no-SVR patients.

## Discussion

This study was designed to collect long-term data on the durability of SVR in patients treated with simeprevir plus PR in Phase II/III studies, and who had achieved SVR at LPVPS. The time for NS3 amino acid substitutions at positions of interest to return to baseline sequence in patients who did not achieve SVR at LPVPS was also assessed. Safety and liver disease evolution were also investigated. No study medication was administered in this follow-up study.

In all SVR patients, SVR was maintained (200/200 [100%]) until the last available visit in the present study (median follow-up time: 35.8 months [range: 6.7–38.4 months]), regardless of any baseline characteristic, including age, the presence of a Q80K polymorphism in GT1a, other NS3 polymorphism, or cirrhosis. Long-term virologic data for DAA/PR regimens are limited and mostly available as congress presentations; however, these results are similar to those shown in a 3-year follow-up study investigating the long-term efficacy of daclatasvir-containing DAA regimens, in which 99.5% (838/842) of patients maintained SVR [15]. Similarly, in the EXTEND study of telaprevir-containing regimens, > 99% of patients maintained SVR over a follow-up period of 21 months [16]. In sofosbuvir-treated patients in the DALTON-C registry study, > 99% of patients maintained SVR over 21 months [17]. In a further follow-up study using registry study data and including sofosbuvir-based and other DAA regimens with or without PR, SVR was maintained in 99.7% of patients treated [18]. Furthermore, patients treated with a DAA (including telaprevir, danoprevir, faldaprevir, simeprevir, mericitabine and balapiravir) in combination with PR were followed for a median of 21 months and 98% maintained SVR [19].

For 48/49 patients enrolled in this study who did not achieve SVR in their simeprevir plus PR parent study, paired baseline and time-of-failure sequencing information was available from the parent study. The majority (43/48 [89.6%]) had emerging amino acid substitutions at NS3 positions 80, 122, 155 and/or 168 at time of failure in the parent study, representative of those observed at time of failure in the overall population of patients not achieving SVR in the Phase IIb/III studies.

At the end of the current study, in 37/43 (86.0%) of enrolled no-SVR patients, emerging NS3 amino acid substitutions were no longer detected by population sequencing (median follow-up time: 179.9 weeks [range: 46.7–230.3 weeks]). The median time to return to baseline sequence was substantially shorter in GT1b- vs GT1a-infected patients (GT1b: 30.5 weeks vs GT1a/other: 67.4 weeks). A total of six patients (three GT1a- and three GT1b-infected patients) still had emerging NS3 emerging amino acid substitutions detectable at the end of this study (5/6 patients completed the study and had follow-up data until Month 36). These six patients had single, or a combination of NS3 amino acid substitutions Q80R, Q80K, R155K and/or D168E, which are substitutions that became undetectable in other patients in this study, and have been previously shown to become undetectable in the majority of patients [20]. Since these four substitutions have been observed in this study (at a low prevalence) as naturally occurring polymorphisms in treatment-naïve and -experienced patients [9], these substitutions are considered to be viable.

These results provide further evidence that emerging NS3 amino acid substitutions become undetectable over time and confirm the data from two previous analyses of the Phase IIb/III simeprevir plus PR studies, which demonstrated that 50.0% and 69.3% of the emerging NS3 amino acid substitutions detected by population sequencing at the time of failure were no longer detected at the end of the studies [9, 20].

Furthermore, similar results were found in an analysis of the Phase III studies of telaprevir plus PR, in which 60.0% of patients with emerging NS3 amino acid substitutions at time of failure had lost their resistance after a median follow-up time of 9.6 months [21]. A similar analysis found that 85% of the patients’ emerging NS3 amino acid substitutions disappeared over time [16]. Although these studies were based on DAA/PR treatment, it is important to note that these findings have also been demonstrated in IFN-free treatment combinations [22, 23].

The impact of persistence of treatment-emergent amino acid substitutions is not fully understood, and NS5A amino acid substitutions can persist for a long time [10]. However, data have shown that patients failing treatment with NS3 or NS5A inhibitors may be successfully re-treated with available regimens, even in the presence of amino acid substitutions [24, 25]. Re-treatment strategies have recently been studied in Phase III trials. The 3-DAA combination of sofosbuvir, velpatasvir and voxilaprevir for 12 weeks was highly effective in patients who failed to respond to prior treatment with DAAs, leading to SVR12 in 99% of non-cirrhotic patients [26].

Hepatic disease progression was assessed as a secondary objective in this study with optional data collection, and the limited data available are displayed in Additional file 1. These data do not allow firm conclusions to be drawn, but are in line with the expectation that less advanced stages of liver disease are associated with higher rates of SVR12.

AEs were reported for a small proportion of patients (4.4%) during the 36-month follow-up period. As expected, no safety findings in the long-term follow-up study were related to simeprevir treatment in the parent studies. In addition, no clinically relevant trends in any laboratory parameters were noted during this follow-up study. Of note, the incidence of new HCC was infrequent in this study (4/249 patients), and a recent meta-analysis concluded that there is no evidence for a higher risk of HCC occurrence or recurrence after DAA treatment [27].

Strengths of this study include its prospective nature, which allowed patients to be followed up for the duration of the study (up to 3 years) to observe outcomes and collect data, and minimized the potential for bias in data generation and statistical analysis. This allowed the assessment of the time taken for emerging NS3/4A amino acid substitutions to return to baseline sequence over a longer time period than in the parent study.

A limitation of this study was that fibrosis assessments were optional and were therefore rarely performed or reported. Accordingly, conclusions on evolution of liver disease stages cannot be made with certainty. In addition, the no-SVR group was small, and patients treated with PR only were not included.

## Conclusions

In conclusion, this 3-year follow-up study provides evidence for the long-term durability of SVR (100%) after successful treatment with simeprevir plus PR for a median of 35.8 months. In the majority (86%) of patients not achieving SVR and with emerging NS3 amino acid substitutions at the time of failure, these amino acid substitutions were no longer detectable at the end of this study after a median follow-up time of 179.9 weeks (41.3 months). No safety concerns were reported. These data are consistent with other studies investigating the long-term outcomes after the treatment of HCV with DAA-containing regimens.

## Additional file

Additional file 1: Results (liver disease evolution) Description of data: Hepatic disease progression was assessed as a secondary objective in this study with optional data collection, and the limited data available are displayed in this additional file. (DOCX 17 kb)

## Abbreviations

AE: Adverse event
DAA: Direct-acting antiviral
EOS: End of study
EU: European Union
GT: Genotype
HCC: Hepatocellular carcinoma
HCV: Hepatitis C virus
HIV: Human immunodeficiency virus
IFN: Interferon
*IL28B*: Interleukin-28b
LLOQ: Lower limit of quantification
LPVPS: Last post-therapy follow-up visit of the parent study
PR: Pegylated interferon and ribavirin
QD: Once-daily
RAS: Resistance-associated substitution
SAE: Serious adverse event
SVR: Sustained virologic response
SVR12: Sustained virologic response 12 weeks after the end of treatment
