# Supplementary material for: A 3-year follow-up study after treatment with simeprevir in combination with pegylated interferon-α and ribavirin for chronic hepatitis C virus infection
Source: Virol J. 2018 Jan 30;15:26. doi: 10.1186/s12985-018-0936-4 (PMC5789677; doi:10.1186/s12985-018-0936-4)
Supplement: Additional file 1: — Results (liver disease evolution) Description of data: Hepatic disease progression was assessed as a secondary objective in this study with optional data collection, and the limited data available are displayed in this additional file. (DOCX 17 kb) [file 12985_2018_936_MOESM1_ESM.docx]

# Additional file 1

# Results

## Liver disease evolution

METAVIR scores were collected during the study on an optional basis. No liver biopsies were required during the study. Therefore, FibroScan measurements were performed and converted to METAVIR scores. Since these assessments were optional, FibroScan data were available from only 38/200 (19.0%) sustained virologic response (SVR) patients. For 30/38 (78.9%) patients with available follow-up results, the fibrosis stage changed over time. Numerically, the METAVIR score decreased in 25/30 (83.3%) patients, indicating an improvement in liver fibrosis, and the METAVIR score increased in 5/30 (16.7%) SVR patients. Follow-up results were available for 18/49 (36.7%) no-SVR patients (measured by FibroScan [72.2%], Biopsy [5.6%], FibroTest [5.6%], acoustic radiation force impulse imaging [5.6%], elastography [5.6%], shear wave elastography [5.6%] and ultrasound elastography, [5.6%]; although seven patients had >1 method used during the study). For 11/18 (61.1%) patients with available follow-up results, the fibrosis stage changed over time. Numerically, the METAVIR score decreased in 3/11 (27.3%) patients and increased in 7/11 (63.6%) patients. In one patient, the METAVIR score decreased from baseline but subsequently returned to the baseline score.
